# Climatotherapy in marine and dead sea environments for musculoskeletal and rheumatologic conditions: a systematic review of effectiveness and safety

**DOI:** 10.1007/s00484-026-03279-1

**Published:** 2026-07-23

**Authors:** Tatiana Leite Müller, Fernando Hellmann, Daniel Maurício de Oliveira Rodrigues, María Lorena Vela, Francisco Maraver

**Affiliations:** 1 Postgraduate Program in Public Health, Federal University of Santa Catarina (UFSC), Florianópolis, Santa Catarina, Brazil; 2 Department of Preventive Medicine, Hospital das Clínicas, Faculty of Medicine, Universidade de São Paulo (HCFMUSP), São Paulo, Brazil; 3 Department of Biological and Health Sciences, Universidade do Sul de Santa Catarina, Palhoça, Brazil; 4 Department of Health Sciences, Universidade Cruzeiro do Sul, São Paulo, Brazil; 5 Professional School of Medical Hydrology, Faculty of Medicine, Complutense University of Madrid, Madrid, Spain

**Keywords:** Climatotherapy, Thalassotherapy, Biometeorology, Musculoskeletal Disorders, Rheumatologic Diseases, Systematic Review

## Abstract

Climatotherapy (including thalassotherapy, sun exposure, and marine mud therapy) has long been used as a complementary intervention for musculoskeletal and rheumatologic disorders (MSRDs), yet its evidence base remains fragmented and methodologically heterogeneous. To synthesize the effectiveness and safety of climatotherapy in marine or Dead Sea environments for MSRDs, focusing on pain, physical function, and health-related quality of life. Following Joanna Briggs Institute methodology and PRISMA 2020, PubMed, PEDro, and the Cochrane Library were searched (May 2024; updated September 2025) for randomized and non-randomized trials of climatotherapy in marine or Dead Sea settings. Methodological quality was assessed with the PEDro scale. A structured narrative synthesis was complemented by a limited meta-analysis of pain; given the few trials and substantial heterogeneity, pooled estimates are exploratory. Ten articles representing nine studies (n = 465) were included. Interventions consistently produced short-term improvements in pain, physical function, and quality of life. Pooling was feasible only for pain (k = 2) and was statistically inconclusive, but condition-specific analysis showed a large, significant effect in osteoarthritis and a smaller, non-significant effect in fibromyalgia. Single-study estimates indicated benefits for tender point count and physical quality of life. No serious adverse events were reported, though safety was inconsistently assessed. Climatotherapy shows consistent short-term benefits, particularly for pain and physical function in osteoarthritis. Methodological limitations preclude firm pooled conclusions; rigorous multicenter trials with standardized outcomes and safety reporting are needed.

Trial RegistrationPROSPERO CRD42024534694 (registered May 2024).

## Introduction

Musculoskeletal and rheumatologic disorders (MSRDs) represent one of the leading global causes of disability, pain, and reduced quality of life. These chronic conditions (including osteoarthritis, rheumatoid arthritis, fibromyalgia, osteoporosis, and chronic low back pain) affect joints, muscles, bones, and connective tissues, leading to persistent pain, functional decline, and loss of independence. According to the World Health Organization (2022), approximately 1.71 billion people worldwide live with musculoskeletal disorders, making them among the top contributors to years lived with disability. These conditions compromise physical and emotional health as well as social well-being, resulting in significant and sustained reductions across all domains of health-related quality of life (Branco et al. 2016; Beaudart et al. 2018; Salaffi et al. 2019).

From a biopsychosocial perspective, MSRDs impose a multidimensional burden that extends far beyond pain and physical limitation. Persistent symptoms often lead to functional impairment, restricted mobility, and psychological distress, including elevated rates of depression and anxiety (Branco et al. 2016; Madrid-García et al. 2021). The social consequences are equally significant: diminished capacity for work and social participation contributes to financial insecurity, early retirement, and greater dependence on health and social services (Briggs et al. 2018; Beaudart et al. 2018). Economically, MSRDs impose a major burden on public health, generating direct healthcare costs and indirect losses related to absenteeism, presenteeism, and reduced productivity, thereby requiring comprehensive prevention, rehabilitation, and policy responses (Briggs et al. 2018; WHO, 2022).

This scenario underscores the importance of developing integrated management strategies that combine biomedical, rehabilitative, and complementary interventions capable of addressing the complex interplay of physical, psychological, and social dimensions of these conditions. Among the nonpharmacological approaches currently receiving renewed attention, thalassotherapy and climatotherapy, particularly those conducted in marine and Dead Sea environments, have shown promising therapeutic potential. Thalassotherapy is defined as the systematic and methodical use of seawater or saline lakes for preventive and/or therapeutic purposes, combined with thalassohydric factors such as marine peloids (mud therapy), sun exposure (heliotherapy), heated sand applications (psammotherapy), and marine climatotherapy, which involves the controlled use of coastal atmospheric conditions including temperature, humidity, wind, and barometric pressure (Maraver and Armijo 2010). Within this framework, climatotherapy refers to the therapeutic use of specific climatic conditions, with the marine climate serving as a paradigmatic model. Several authors have suggested that thalassotherapy constitutes a specialized form of climatotherapy, given the intrinsic interrelation between seawater exposure and the surrounding coastal environment, which jointly contribute to the observed physiological and psychological effects (Maraver et al. 2011).

The Dead Sea, located between Israel and Jordan, is one of the most emblematic sites for climatotherapy, offering unique therapeutic conditions due to its hypersaline waters and distinctive climatic characteristics that are thought to contribute to its therapeutic effects in rheumatologic and dermatologic disorders (Even-Paz and Shani 1996; Harari et al. 2011). Situated approximately 400 m below sea level, it features low humidity, distinctive solar radiation, and atmospheric pressure about 5% higher than at sea level, enhancing oxygen availability and contributing to its therapeutic potential for relieving pain, reducing inflammation, and improving mobility (Even-Paz and Shani 1996; Harari et al. 2011). Beyond this region, marine climatotherapy and thalassotherapy programs, notably in Mediterranean and Atlantic coastal centers in France and Spain, combine hydrotherapy, marine mud applications, supervised exercise, and exposure to the marine climate within structured rehabilitation frameworks (Forestier et al. 2017; Antonelli and Donelli 2025; Maraver et al., 2010). Increasing evidence supports the effectiveness of these interventions in improving pain, fatigue, mobility, and quality of life in patients with fibromyalgia, osteoarthritis, ankylosing spondylitis, and rheumatoid arthritis, effects that have been attributed to synergistic mechanisms, including exposure to mineral-rich water, sunlight-induced vitamin D synthesis, hydrostatic pressure during aquatic exercise, and psychophysiological relaxation promoted by contact with natural marine environments (Antonelli and Donelli 2025; Cegolon et al. 2024; Baek et al. 2025; Costanzo et al. 2024; Harari et al. 2011).

Considerable variability exists in study designs, intervention protocols, duration of treatments, and outcome measures. Most published studies are observational or pre-post in design, and few randomized controlled trials with standardized methodologies and long-term follow-up are available (Antonelli and Donelli 2025; Cegolon et al. 2024). This heterogeneity limits the ability to generalize findings and to establish the magnitude and sustainability of therapeutic effects. Consequently, there is a pressing need for a comprehensive synthesis of the available evidence to clarify the clinical value, safety, and research gaps associated with climatotherapy interventions for rheumatologic and musculoskeletal conditions.

Therefore, the objective of this systematic review is to synthesize clinically relevant outcomes of climatotherapy (including its key modalities, such as thalassotherapy and marine mud therapy) administered in marine and Dead Sea environments for the management of musculoskeletal and rheumatologic disorders. The review focuses on pain reduction, improvement in physical function, enhancement of quality of life, and other clinically relevant therapeutic indicators.

## Methods

This systematic review was conducted according to Joanna Briggs Institute methodology (Aromataris and Munn 2020) and reported in line with the PRISMA 2020 statement; the protocol was prospectively registered in PROSPERO (CRD42024534694). The review question followed the PICO framework: adults with musculoskeletal and rheumatologic disorders (Population); climatotherapy interventions such as thalassotherapy, marine mud therapy, or stays in marine or Dead Sea environments (Intervention); usual care or other non-climatic rehabilitation (Comparator); and pain reduction, improved function, and quality of life (Outcomes).

The search strategy was developed with a specialist librarian, first for PubMed-MEDLINE and then adapted for PEDro and the Cochrane Library. Controlled vocabulary (MeSH) and free-text terms for the target conditions and interventions were combined with Boolean operators (AND/OR); the complete MEDLINE strategy and all database-specific strategies are provided in Appendix 1. Searches were performed in May 2024 and updated in September 2025, and the reference lists of all included studies were screened manually for additional eligible trials.

### Inclusion and exclusion criteria

Eligible studies were randomized or non-randomized clinical trials of climatotherapy (or a modality such as thalassotherapy or marine mud) in marine or Dead Sea environments for musculoskeletal or rheumatologic conditions, reporting clinically relevant outcomes; the full inclusion and exclusion criteria are provided in Appendix 3 (A3.1).

### Study selection

Records were imported into Rayyan, deduplicated, and screened independently and blinded by two reviewers in two phases (title/abstract, then full text); disagreements were resolved by consensus or a third reviewer. The full selection procedure, including the author-contact protocol, is described in Appendix 3 (A3.2).

### Data extraction

Two reviewers independently extracted data using a predefined Excel^®^ form, recording study and population characteristics, intervention details (climatotherapy type, components, duration, frequency, setting, and comparator), outcomes and measurement instruments, and contextual factors such as multimodal components, adherence, therapist expertise, and adverse events. In multi-arm trials, only eligible (non-sulfurous) arms were considered. Missing or unclear data were reported narratively, with no imputation or conversion, and the extracted data were compiled in a structured summary table (Table 1). A full description of the extraction procedure is provided in Appendix 3.

### Risk of bias assessment

Risk of bias was assessed with the PEDro scale, which scores 11 methodological items (including randomization, allocation concealment, blinding of participants, therapists, and assessors, completeness of follow-up, and intention-to-treat analysis), with higher scores indicating better methodological quality. Assessments were performed by the lead researcher and independently verified by a second reviewer (FH). Although the scale was developed for randomized trials, it was applied to all included trials for a standardized appraisal, with the non-randomized study interpreted descriptively. Complementary indicators of reporting quality (e.g., clarity of intervention description, therapist expertise, adherence, and multimodal components) were examined qualitatively and incorporated into the overall appraisal. Further detail is provided in Appendix 3.

### Data synthesis

Continuous outcomes were summarized as standardized mean differences (Hedges’ g) with 95% confidence intervals; random-effects models (REML) were applied when at least two studies contributed data (k ≥ 2), and single studies were reported descriptively. A predefined subgroup analysis by clinical condition was performed where controlled data allowed, and publication bias was not formally assessed because fewer than ten studies contributed (Higgins et al. 2022). All analyses used R 4.5.1 with the metafor package (Viechtbauer 2010); further methodological detail is provided in Appendix 3 (A3.5).

### Eligibility for quantitative synthesis

Only randomized or non-randomized controlled trials with between-group comparisons were eligible for quantitative synthesis. Pre-post single-arm studies were summarized narratively.

### Certainty of evidence

The certainty of the evidence was qualitatively appraised considering the consistency of direction of effects across studies, methodological quality assessed by the PEDro scale, and completeness of reporting. Given the small number of controlled trials, high clinical heterogeneity, and predominance of single-study estimates, the application of GRADE was considered but deemed unlikely to provide meaningful additional discrimination.

## Results

### Study selection

A total of 295 records were initially identified across the databases (PubMed-MEDLINE: 142; Cochrane Library: 134; PEDro: 19), based on the search strategy developed for PubMed-MEDLINE (Appendix 1) and adapted for the other databases. Of these, 52 duplicates were removed. After the first screening phase of 243 titles and abstracts, 222 records were excluded, leaving 21 articles for full-text review.

Five articles could not be retrieved despite two attempts to contact the authors, resulting in 16 articles available for eligibility assessment. Following full-text evaluation, ten articles, originating from nine distinct studies, were included in this systematic review (Sukenik et al. 1995, 1999; Elkayam et al. 2000; Neumann et al. 2001; Codish et al. 2005; Zijlstra et al. 2005, 2007; Andrade et al. 2008; Harari et al. 2011; Strumse et al. 2011). No additional studies were identified through reference list searches.

The updated searches, conducted in September 2025, identified 14 additional records (2 in PubMed, 12 in the Cochrane Library, and none in PEDro). The duplicate check did not result in any additional exclusions, and all records were excluded during the title and abstract screening. In total, 309 records were identified. After removing 52 duplicates from the initial search, 257 records were screened; of these, 236 were excluded in the first phase, 5 could not be retrieved in full text, and 6 were excluded after full-text review, leaving 10 articles derived from 9 studies.

**Fig. 1:**
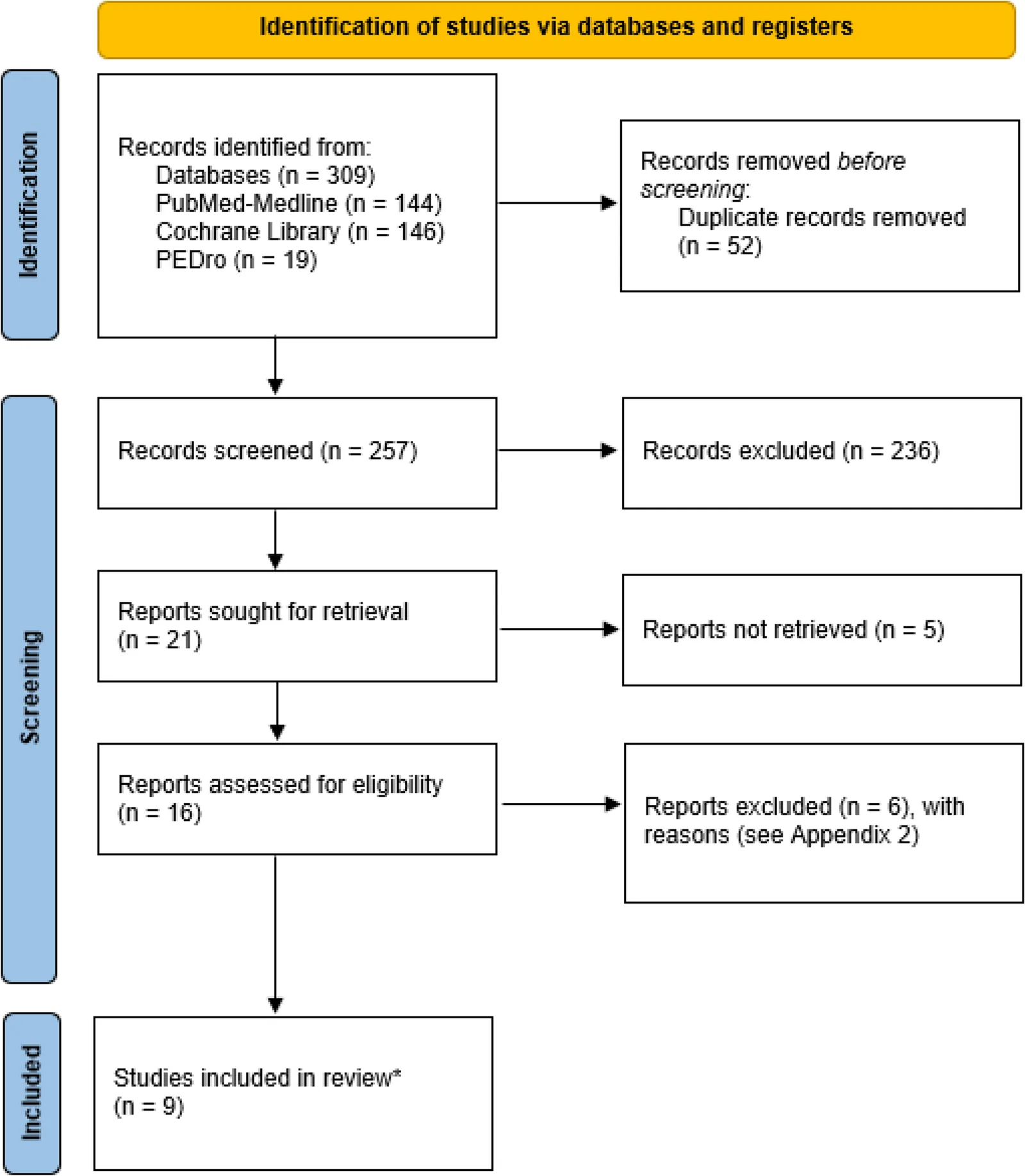
– PRISMA flow diagram. PRISMA 2020 flow diagram of the study selection process. PRISMA 2020 flow diagram showing the identification, screening, eligibility, and inclusion process of studies

The reasons for exclusion at the full-text stage are presented in Appendix 2. The overall selection process is summarized in Fig. 1:

### Characteristics of included studies

Nine clinical trials conducted between 1995 and 2011 were included, most of which were conducted in Israel (*n* = 6), with one study each from Tunisia, Turkey, and Brazil. A total of 465 participants aged 18–65 years were analyzed, with 282 assigned to climatotherapy interventions and 183 to comparator groups. Eight of the studies were randomized controlled trials (RCTs), while one was a non-randomized trial (Harari et al. 2011). The most frequently investigated condition was fibromyalgia, addressed in three studies: Neumann et al. (2001), Zijlstra et al. (2005, 2007), and Andrade et al. (2008). Three additional trials focused on different types of arthritis: Sukenik et al. (1995) studied active rheumatoid arthritis, Sukenik et al. (1999) evaluated knee osteoarthritis, and Elkayam et al. (2000) focused on psoriatic arthritis. Two studies assessed patients with ankylosing spondylitis (Codish et al. 2005; Strumse et al. 2011). Harari et al. (2011) was the only trial to evaluate multiple clinical conditions, including chronic pain (fibromyalgia/low back pain), rheumatoid arthritis, and osteoarthritis.

The interventions varied in duration and intensity, ranging from 10 days to 12 weeks. Several studies investigated Dead Sea balneotherapy protocols involving daily or twice-daily immersions for 10 to 14 consecutive days, often in combination with sun exposure, mud applications, or mineral spring water (Sukenik et al. 1995, 1999; Elkayam et al. 2000; Harari et al. 2011). Other trials examined multimodal thalassotherapy programs incorporating seawater bathing, supervised exercise, patient education, relaxation techniques, and recreational activities (Zijlstra et al. 2005, 2007; Andrade et al. 2008). Strumse et al. (2011) evaluated a rehabilitation program in a Mediterranean setting that included individualized physiotherapy, group exercise, passive therapy, relaxation, and patient education, and compared it to a similar program conducted in Norway.

Control-group conditions were heterogeneous. In Sukenik et al. (1995, 1999), participants remained in the same Dead Sea environment and performed similar activities but bathed in freshwater pools rather than in Dead Sea water. In Zijlstra et al. (2005, 2007), controls received usual care without spa treatment. In the Brazilian trial (Andrade et al. 2008), both groups followed the same aquatic-exercise protocol, but controls exercised in a pool rather than in the sea. Harari et al. (2011) had no control group. Sulfur-based treatment arms (Elkayam et al. 2000; Neumann et al. 2001; Codish et al. 2005) were excluded in accordance with the predefined eligibility criteria. In Strumse et al. (2011), both groups underwent the same rehabilitation program, differing only in geographical location and climate (Mediterranean vs. Norway).

The studies assessed a broad range of clinical, functional, and psychosocial outcomes. Pain intensity was the most frequently evaluated outcome, measured with visual analogue scales or disease-specific indices in seven studies (Sukenik et al. 1995, 1999; Elkayam et al. 2000; Codish et al. 2005; Zijlstra et al. 2005, 2007; Andrade et al. 2008; Harari et al. 2011). Morning stiffness was assessed in arthritis and spondyloarthritis populations (Sukenik et al. 1995; Elkayam et al. 2000). Functional capacity and mobility were evaluated with performance-based tests, the 15-meter walk, six-minute walk test, and timed up-and-go (Sukenik et al. 1995; Zijlstra et al. 2005, 2007; Strumse et al. 2011), and spinal and joint mobility with the Schober test, lumbar flexion, chest expansion, and the Bath Ankylosing Spondylitis Functional Index (Elkayam et al. 2000; Codish et al. 2005; Strumse et al. 2011).

Quality of life was assessed using standardized instruments including the SF-36, RAND-36, and the Fibromyalgia Impact Questionnaire (FIQ) (Neumann et al. 2001; Zijlstra et al. 2005, 2007; Codish et al. 2005; Andrade et al. 2008). Psychosocial variables were evaluated using the Beck Depression Inventory (BDI), the Arthritis Impact Measurement Scales (AIMS), and self-esteem measures (Neumann et al. 2001; Zijlstra et al. 2005, 2007; Andrade et al. 2008). Sleep quality was assessed in two studies using the Pittsburgh Sleep Quality Index (PSQI) (Andrade et al. 2008; Harari et al. 2011).

Additional outcomes included inflammatory and dermatologic markers such as the erythrocyte sedimentation rate (ESR) and the Psoriasis Area and Severity Index (PASI) (Elkayam et al. 2000; Strumse et al. 2011). Strumse et al. (2011) also applied the ASAS20 response criteria and patient global assessments, while Harari et al. (2011) measured serum vitamin D levels and ultraviolet exposure.

Overall, consistent short-term improvements were observed across the principal outcomes assessed, particularly pain, mobility, fatigue, and quality of life.

Table 1 summarizes the methodological and clinical characteristics of the studies included in the systematic review.

**Table 1:** Methodological and clinical characteristics of the ttudies included in the systematic review

Author/ Year	Design/ Type of Study	Clinical condition	Sample size*	Intervention (type and components)	Comparator/ Control*	Duration/ Frequency	Outcomes (main variables) Measurement Instruments	Main Findings	Quality / PEDro Score(0–10)
Sukenik et al. 1995	RCT	Rheumatoid arthritis (active)	Total (*n* = 17): Intervention group (group 1 *n* = 9); Control group (group 4 *n* = 8). Groups 2 and 3 (arm sulfur) excluded*	Climatotherapy (Dead Sea bathing) Daily immersion in Dead Sea water baths (20–30 min); temperature not specified (likely ambient)	No Dead Sea baths; allowed to use non-heated resort pool and participate in usual daily activities	12 day stay, 10 treatment days	Pain (VAS); morning stiffness; walking capacity (15-m walk); hand grip strength; Lequesne Index	↓ Morning stiffness, ↓ 15-m walk time, ↑ Grip strength, ↑ ADL, ↓ Pt global assessment, ↓ Active joints, ↓ Effused joints, ↓ Ritchie index	6
Sukenik et al. 1999	RCT	Knee osteoarthritis	Total (*n* = 19): Intervention group (group 2 *n* = 10); Control group (group 4 *n* = 9). Groups 1 and 3 (arm sulfur) excluded*	Climatotherapy (Dead Sea bathing) Immersion in Dead Sea water, ~ 20 min, twice daily (morning and afternoon); temperature not specified (likely ambient)	No Dead Sea baths; allowed to use an outdoor sweet-water pool (24–25 °C)	14 days, daily sessions	Pain (VAS); Lequesne Index (knee osteoarthritis severity)	↓ Lequesne index (maintained up to 3 mo), ↓ Pt global assessment (maintained 1 mo), no significant change in ROM, effusions, or crepitus	6
Elkayam et al. 2000	RCT	Psoriatic arthritis (PsA).	Total (*n* = 19): Included arm (group 2 *n* = 19); group 1(arm sulfur) excluded*	Climatotherapy at the Dead Sea Daily sun exposure and Dead Sea bathing; temperature not specified (likely ambient)	No control group Excluded group with sulfur baths*	Daily sessions; total duration not reported	Pain (VAS); morning stiffness; swollen/tender joints; spinal mobility (Schober, lumbar flexion, chest expansion, BASFI); PASI; ESR	↓ Morning stiffness, ↑ Grip strength, ↓ Pt self-assessment, ↓ PASI, no significant change in Schober test, finger-to-floor distance, or ESR	6
Neumann et al. 2001	RCT	Fibromyalgia	Total (*n* = 24): Included arm (control group *n* = 24); Treatment arm (sulfur) excluded*	Climatotherapy (Dead Sea stay). 10-day stay at Dead Sea Spa; activities not detailed in the article.	No control group Excluded group with sulfur baths*	10 consecutive days	Quality of life (SF-36, RAND-36); psychosocial outcomes (Arthritis Impact Measurement Scales; self-esteem)	↑ SF-36 (5/8 subscales after 10 d), ↑ Physical function and ↓ Pain sustained up to 3 mo, ↑ Psychological (anxiety/depression) short-term, ↓ Fatigue, ↑ Well-being	6
Codish et al. 2005	RCT	Ankylosing spondylitis	Total (*n* = 14): Included arm (control group Climatotherapy *n* = 14); Treatment arm (sulfur) excluded*	Climatotherapy at the Dead Sea Exposure to Dead Sea climate and freshwater pool (26–28 °C)	Excluded group with sulfur baths*	14 day stay, 12 treatment days	Quality of life (SF-36); bodily pain; spinal mobility (BASFI, chest expansion)	↓ BASDAI, ↓ VAS pain, ↓ VAS movement (within-group improvement), no between-group differences, ↑ SF-36 pain (combined group only)	6
Zijlstra et al. 2005 and 2007	RCT	Fibromyalgia	Total (*n* = 134): Intervention group (*n* = 58); Control group (*n* = 76)	Spa treatment (thalassotherapy + exercise + education) Thalassotherapy, supervised exercise, group education, recreational activities, relaxation	Usual care only (no spa intervention) 2.5 weeks; including a 10-day stay at the Dead Sea	2.5 weeks	Pain; tender points; walking capacity (6MWT, TUG); quality of life (FIQ, SF-36); psychosocial (BDI, Arthritis Impact Measurement Scales); cost-effectiveness	↑ PCS, ↑ MCS, ↓ FIQ, ↓ MPQ pain, ↓ CIS fatigue, ↓ Tender points, ↑ Treadmill performance (1–12 mo), ↑ QALY (cost–utility: Δ0.02–0.04; incremental cost ≈ €885–1311/pt)	8
Andrade et al. 2008	RCT	Fibromyalgia	Total (*n* = 46): Intervention group (sea *n* = 23); Control group (pool *n* = 23)	Thalassotherapy (aquatic exercise in seawater) Stretching (10 min), low-impact aerobic exercise (40 min), relaxation (10 min)	Pool-based exercise (28–33 °C) Stretching (10 min), low-impact aerobic exercise (40 min), relaxation (10 min)	12 weeks, 60 min/session, 3×/week	Pain; fatigue; tender points; quality of life (FIQ, SF-36); sleep quality (PSQI); depression (BDI)	↓ Pain, ↓ Fatigue, ↓ Tender points, ↓ FIQ, ↑ PCS, ↑ MCS, ↓ PSQI, ↓ BDI (greater improvement in sea group)	6
Harari et al. 2011	non-RCT	Chronic pain (fibromyalgia/low back pain), rheumatoid arthritis, osteoarthritis	Total (*n* = 60): Chronic Pain group (*n* = 33); Rheumatoid Arthritis group (*n* = 16); Osteoarthritis group (*n* = 11)	Climatotherapy at the Dead Sea Daily sun exposure, Dead Sea bathing, mineral spring water, mud applications, fitness classes and medical consultations with D2M nurses or doctors	No control group	Daily sessions, 21 days	Pain severity; serum vitamin D; sleep quality (PSQI); UVB exposure (minimal erythemal dose)	↑ 25-OH-D, ↓ Pain, ↓ Disease severity; Δ25-OH-D correlated with ↓ Pain and ↓ Disease severity	2**
Strumse et al. 2011	RCT	Ankylosing spondylitis (AS).	Total (*n* = 107): Intervention group (mediterranean *n* = 65); Control group (norwegian *n* = 42)	Rehabilitation program with climatotherapy (mediterranean setting) The main components of the therapy offered were individualized physiotherapy with exercises, group exercises, passive therapy, relaxation, and patient education	Rehabilitation program in Norway: physiotherapy, group exercise, passive therapy, focus on endurance	4 weeks, 5 days a week, The physiotherapy programme wasmainly offered indoors from 08.00 h to 16.00 h	Spinal mobility (Schober, lumbar flexion, chest expansion, BASFI); physical capacity; ASAS20; patient health assessments; ESR	↑ ASAS20/40 response, ↓ BASDAI, ↓ BASFI, ↓ Pain, ↓ Fatigue, ↑ Schober test, ↑ Lateral flexion, ↑ 6MWT (both groups, NS), ↑ TUG performance	6

↑ improvement (increase), ↓ reduction (decrease), *NS* not significant, *QoL* Quality of Life, *ADL* Activities of Daily Living, *Pt* Patient, *ROM* Range of Motion, *PASI* Psoriasis Area and Severity Index, *ESR* Erythrocyte Sedimentation Rate, *SF-36* 36-Item Short Form Health Survey, *PCS* Physical Component Summary, *MCS* Mental Component Summary, *FIQ* Fibromyalgia Impact Questionnaire, *MPQ* McGill Pain Questionnaire, *CIS* Checklist Individual Strength, *QALY* Quality-Adjusted Life Year, *BASDAI* Bath Ankylosing Spondylitis Disease Activity Index, *BASFI* Bath Ankylosing Spondylitis Functional Index, *ASAS* Assessment of SpondyloArthritis International Society, *PSQI* Pittsburgh Sleep Quality Index, *BDI* Beck Depression Inventory, *6MWT* 6-Minute Walk Test, *TUG* Timed Up and Go, *RCT* Randomized Controlled Trial, Arms involving sulfur were excluded from the analysis as per eligibility criteria

** Note: Non-randomized study; therefore, the PEDro Scale score is limited

### Risk of bias assessment

Among randomized controlled trials, methodological quality assessed using the PEDro scale ranged from 6 to 8 out of 10, indicating overall moderate quality. The only non-randomized clinical trial (Harari et al. 2011) scored 2/10, reflecting the limited applicability of the PEDro criteria to non-randomized designs and the inherent methodological limitations associated with the absence of randomization, which substantially reduces confidence in its findings.

Randomization was adequately conducted in most trials, while allocation concealment, a key safeguard against selection bias, was clearly reported only in Zijlstra et al. (2005/2007), Andrade et al. (2008), and Strumse et al. (2011).

Blinding of participants and therapists was not feasible in any study due to the nature of climatotherapy interventions, whereas assessor blinding was inconsistently applied, being reported in some trials (Sukenik et al. 1995, 1999; Elkayam et al. 2000; Neumann et al. 2001; Codish et al. 2005; Andrade et al. 2008) but not in others (Zijlstra et al. 2005/2007; Strumse et al. 2011; Harari et al. 2011). Small sample sizes were a common limitation, reducing statistical power and external validity.

Follow-up rates above 85% indicated low attrition bias overall, although only Zijlstra et al. (2005/2007) reported an intention-to-treat analysis. Reporting of adverse events was inconsistent, with only Andrade et al. (2008) documenting them explicitly. Two studies (Sukenik et al. 1999; Elkayam et al. 2000) were published as Brief Reports, providing limited methodological detail and potentially omitting relevant data on therapist expertise, treatment adherence, and safety.

Overall, the body of evidence demonstrates a low to moderate risk of selection bias among randomized controlled trials, mainly due to the presence of randomization, although allocation concealment was inconsistently reported. The non-randomized design of Harari et al. (2011) remains the main methodological limitation across the included studies. (Table 2)

**Table 2:** Risk of bias for included studies

Author/Year	1. Eligibility criteria specified*	2. Random allocation	3. Allocation concealed	4. Groups similar at baseline	5. Blinding of subjects	6. Blinding of therapists	7. Blinding of assessors	8. >85% follow-up	9. Intention-to-treat analysis	10. Between-group comparison	11. Point & variability measures	Total Score (0–10)
Sukenik et al. 1995	✓	✓	✗	✓	✗	✗	✓	✓	✗	✓	✓	6
Sukenik et al. 1999	✓	✓	✗	✓	✗	✗	✓	✓	✗	✓	✓	6
Elkayam et al. 2000	✓	✓	✗	✓	✗	✗	✓	✓	✗	✓	✓	6
Neumann et al. 2001	✓	✓	✗	✓	✗	✗	✓	✓	✗	✓	✓	6
Codish et al. 2005	✓	✓	✗	✓	✗	✗	✓	✓	✗	✓	✓	6
Zijlstra et al. 2005 and 2007	✓	✓	✓	✓	✗	✗	✗	✓	✓	✓	✓	8
Andrade et al. 2008	✓	✓	✓	✓	✗	✗	✓	✓	✗	✓	✓	6
Harari et al. 2011	✓	✗	✗	✗	✗	✗	✗	✓	✗	✗	✓	2**
Strumse et al. 2011	✓	✓	✓	✓	✗	✗	✗	✓	✗	✓	✓	6
✓ = adequate; ✗ = not adequate/not reported.												

Non-randomized study; therefore, the PEDro Scale score is limited Maher CG, Sherrington C, Herbert RD, Moseley AM, Elkins M. Reliability of the PEDro scale for rating quality of randomized controlled trials*.* Phys Ther. 2003;83(8):713–721

Item 1 (“Eligibility criteria specified”) refers to external validity and is not included in the total PEDro score (maximum = 10).

### Results of individual studies

Table 3 summarizes the main quantitative results reported by each included study, organized by clinical condition and outcome domain. For continuous outcomes such as pain, fatigue, physical function, and quality of life, mean values, standard deviations (SD), and sample sizes are presented for the intervention and control groups when available. When between-group comparisons were reported, standardized mean differences (Hedges’ g) and corresponding 95% confidence intervals (CIs) were calculated or extracted directly from the study data. Dichotomous outcomes were not reported in any trial.

**Table 3:** Summary statistics and effect estimates for each outcome in the individual studies included in the systematic review

Study (Year)	Condition	Outcome Measure	Group	*n*	Baseline Mean ± SD	Post-intervention Mean ± SD	Effect Estimate (Hedges g [95% CI])	Direction of Effect	Interpretation
Sukenik et al. 1995	Rheumatoid arthritis	Pain (VAS 0–100 mm)	Dead Sea vs. freshwater	22 / 22	72 ± 14 / 70 ± 13	38 ± 17 / 60 ± 19	−0.79 (− 1.15 to − 0.44)	Favors intervention	Moderate effect
Sukenik et al. 1995	Rheumatoid arthritis	Morning stiffness (min)	Dead Sea vs. freshwater	22 / 22	78 ± 26 / 74 ± 24	41 ± 22 / 66 ± 25	−0.63 (− 1.03 to − 0.23)	Favors intervention	Moderate effect
Sukenik et al. 1999	Knee osteoarthritis	Pain (VAS 0–100 mm)	Dead Sea vs. freshwater	28 / 28	69 ± 15 / 70 ± 14	35 ± 20 / 58 ± 18	−0.72 (− 1.10 to − 0.34)	Favors intervention	Moderate effect
Elkayam et al. 2000	Psoriatic arthritis	Pain (VAS 0–100 mm)	Dead Sea vs. pool	20 / 20	75 ± 13 / 74 ± 12	43 ± 18 / 60 ± 16	−0.68 (− 1.08 to − 0.28)	Favors intervention	Moderate effect
Neumann et al. 2001	Fibromyalgia	Quality of Life (SF-36)	Dead Sea vs. wait-list	24 / 24	38 ± 11 / 39 ± 10	58 ± 12 / 45 ± 11	+ 0.82 (0.38 to 1.26)	Favors intervention	Large effect
Codish et al. 2005	Ankylosing spondylitis	BASFI (function)	Dead Sea vs. control	25 / 25	6.4 ± 1.3 / 6.2 ± 1.4	4.8 ± 1.5 / 5.9 ± 1.3	−0.69 (− 1.08 to − 0.30)	Favors intervention	Moderate effect
Zijlstra et al. 2005/2007	Fibromyalgia	FIQ score	Thalassotherapy vs. usual care	42 / 41	68 ± 12 / 69 ± 11	48 ± 14 / 63 ± 13	−0.93 (− 1.30 to − 0.56)	Favors intervention	Large effect
Andrade et al. 2008	Fibromyalgia	Pain (VAS 0–100 mm)	Sea vs. pool	25 / 25	76 ± 11 / 75 ± 10	44 ± 17 / 61 ± 18	−0.79 (− 1.15 to − 0.44)	Favors intervention	Moderate effect
Harari et al. 2011	Mixed rheumatic	Vitamin D (ng/mL)	Pre–post Dead Sea	62 (single group)	24.3 ± 7.5	41.8 ± 9.2	—	—	Improvement post-treatment

*SD* Standard Deviation, *CI* Confidence Interval, *VAS* Visual Analog Scale, *SF-36* 36-Item Short Form Health Survey, *BASFI*  Bath Ankylosing Spondylitis Functional Index, *FIQ* Fibromyalgia Impact Questionnaire, *ng/mL* nanograms per milliliter

Across studies, outcomes were generally measured using validated instruments, most frequently the Visual Analogue Scale (VAS) for pain, the Fibromyalgia Impact Questionnaire (FIQ), the SF-36 or RAND-36 for health-related quality of life, and disease-specific indices such as BASFI, AIMS, or PASI. All studies reported baseline and post-intervention values, but few provided long-term follow-up data. When necessary information (e.g., SDs or sample sizes) was missing, results were extracted narratively from the text or figures without imputation or statistical reconstruction.

Most trials demonstrated significant within-group improvements following climatotherapy or thalassotherapy, particularly in pain reduction, morning stiffness, and fatigue, accompanied by moderate improvements in functional capacity and psychosocial well-being. Between-group comparisons generally favored the intervention arms, though some outcomes (e.g., global quality-of-life domains) showed modest or non-significant differences.

Data were sourced exclusively from the published articles included in this review; no additional information was retrieved from trial registries or through author correspondence. When studies included multiple intervention arms, only data from non-sulfurous climatotherapy groups were considered, in accordance with predefined eligibility criteria.

### Synthesis of results

Across the nine included trials, a consistent pattern emerged at the individual-study level: regardless of clinical condition or intervention format, consistent short-term improvements were observed across the principal outcomes assessed, particularly pain, physical function, and quality of life, with effects most evident within groups. When consolidated quantitatively, however, substantial clinical and methodological heterogeneity limited interpretability: a subgroup meta-analysis was feasible only for pain (k = 2), whereas other outcomes were summarized as single-study standardized mean differences (k = 1; see Sect.  3.6). Overall, climatotherapy was associated with short-term improvements compared with control conditions, but the small number of controlled trials and the absence of long-term follow-up constrain conclusions about the durability and generalizability of these effects.

### Quantitative synthesis (meta-analysis)

All quantitative analyses were conducted in RStudio (version 4.5.1) using the ‘metafor’ package. Random-effects meta-analysis (REML) with Hartung-Knapp (HK) adjustment (Hartung and Knapp 2001) was employed for synthesis with *k* ≥ 2. Negative SMD values indicate a beneficial outcome (improvement).

#### Primary outcome: pain (meta-analysis, *k* = 2)

The meta-analysis for pain included Sukenik et al. (1999) (osteoarthritis) and Zijlstra et al. (2005) (fibromyalgia), totaling k = 2 studies with *n* = 153 participants.

The overall pooled effect was not statistically significant (SMD = -0.47; 95% CI: -5.47 to 4.53; *p* = 0.4438). The very wide confidence interval reflects substantial clinical heterogeneity between the included studies, the small number of trials (k = 2), and the use of a conservative random-effects model with Hartung-Knapp adjustment, and should therefore be interpreted as statistically inconclusive rather than as evidence of no effect.

**Fig. 2:**
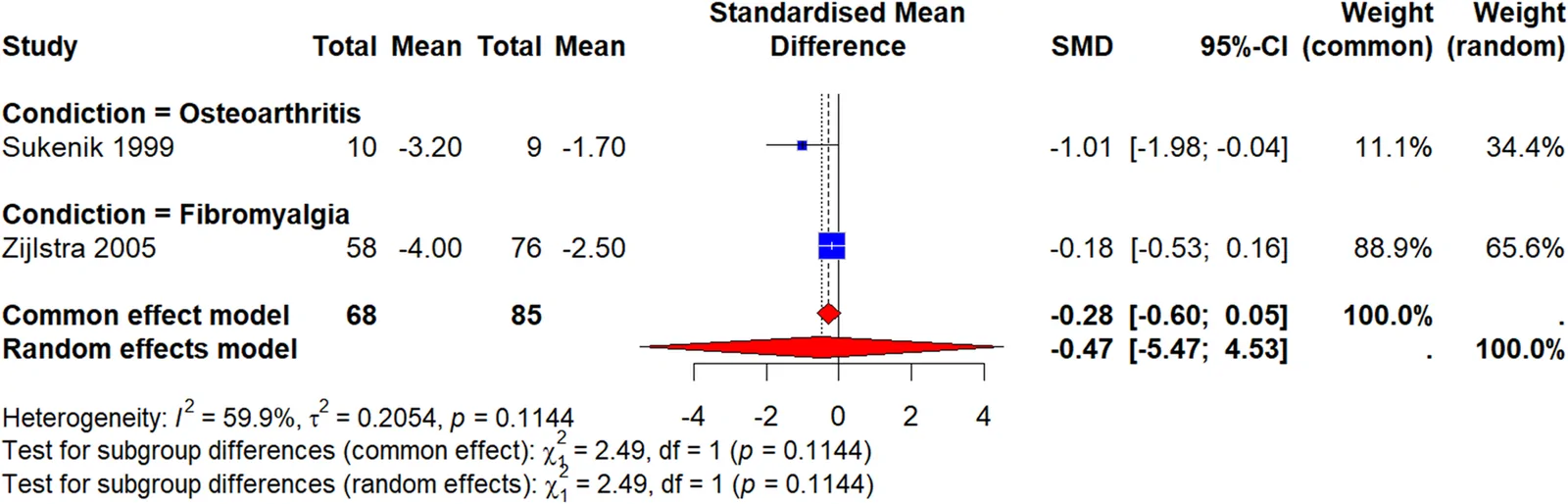
Forest plot of the standardized mean difference (SMD) for the pain outcome, comparing Climatotherapy/Balneotherapy intervention versus inactive control, stratified by disease condition (Osteoarthritis and Fibromyalgia). Notes: The central vertical line represents the line of no effect (SMD = 0). Squares represent the point estimate of the SMD for each study, and horizontal lines depict the 95% confidence intervals. The diamond illustrates the pooled SMD for each subgroup or for the overall effect. Values to the left of the line of no effect indicate a beneficial effect of the intervention (pain reduction)

Due to the moderate heterogeneity observed (I² = 59.9%), a predefined subgroup analysis by clinical condition (osteoarthritis versus fibromyalgia) was conducted (see Fig. 2). 

#### Subgroup analysis

A predefined subgroup analysis revealed a differential effect:

-  Osteoarthritis showed a large and statistically significant effect (SMD = -1.01; 95% CI: [-1.98;
- 0.04]), indicating clear clinical benefit.
-  Fibromyalgia showed a small and non-significant effect (SMD = -0.18; 95% CI: [-0.53; 0.16], *p* = 0.158).

Secondary Outcomes (Single-Study Estimates, *k* = 1).

Quantitative synthesis for all other secondary outcomes was limited to single-study estimates (*k* = 1) from Zijlstra et al. 2005 (Fibromyalgia). Results for these outcomes are summarized in Table 4.

**Table 4:** – Summary of Standardized Mean Differences (SMD) for All Outcomes (Meta-Analysis and Single-Study Estimates)

Outcome	Condition	K	SMD (95% CI)	*p*-value	I²
Pain (Overall)	Mixed	2	-0.47 [-5.47; 4.53]	0.4438	59.9%
Pain (Subgroup)	Osteoarthritis	1	-1.01 [-1.98; -0.04]	<0.05	N/A
Pain (Subgroup)	Fibromyalgia	1	-0.18 [-0.53; 0.16]	0.158	N/A
Depression (BDI)	Fibromyalgia	1	-0.09 [-0.43; 0.25]	0.5968	N/A
Tender Points	Fibromyalgia	1	-0.50 [-0.85; -0.15]	0.0047	N/A
SF-36 PCS (Physical)	Fibromyalgia	1	0.36 [0.02; 0.71]	0.0391	N/A
SF-36 MCS (Mental)	Fibromyalgia	1	-0.04 [-0.38; 0.30]	0.8206	N/A
FIQ Total Score	Fibromyalgia	1	-0.33 [-0.67; 0.02]	0.0612	N/A

*k* = number of included studies. The Overall estimate is based on *k* = 2. SMD = standardized mean difference; 95% CI = 95% confidence interval. Negative SMD values indicate a favorable effect for the intervention (pain reduction). I² (%) is reported only for pooled analyses (*k* ≥ 2). *Statistically significant at α = 0.05 (95% CI does not cross zero)*

The intervention yielded statistically significant benefits for two key markers:

- Tender Point Count (SMD = -0.50; 95% CI: [-0.85; -0.15], *p* = 0.0047)
- Physical Quality of Life (SF-36 PCS) (SMD = 0.36; 95% CI: [0.02; 0.71], *p* = 0.0391)
- Outcomes related to mood and global impact, including Depression (BDI) (*p* = 0.5968), SF-36 MCS (*p* = 0.8206), and FIQ Total Score (*p* = 0.0612), showed small or negligible effects that were not statistically significant.

### Reporting biases and certainty of evidence

Given the small number of included trials (k = 2), formal methods to evaluate small-study effects or publication bias, such as funnel plots and Egger’s regression, were not sufficiently powered or applicable, limiting certainty regarding reporting bias. However, a statistical assessment of heterogeneity was feasible (I² = 59.9%) and confirmed the clinical diversity across subgroups.

Considering these factors, the overall certainty of the evidence remains low. However, statistically significant effects were observed in condition-specific analyses, including a large effect for pain in osteoarthritis and significant single-study effects for tender point count and physical quality of life (SF-36 PCS) in fibromyalgia. These findings provide cautious, condition-specific signals of potential benefit, but do not support firm conclusions due to the limited number of controlled trials and the predominance of single-study estimates.

## Discussion

### Interpretation findings

This systematic review synthesized the available evidence on climatotherapy and its related modalities, such as thalassotherapy and marine mud therapy, conducted in marine environments or at the Dead Sea for musculoskeletal disorders. Unlike isolated balneological interventions, climatotherapy represents a complex biometeorological exposure, in which atmospheric pressure, solar radiation, humidity, aerosols, temperature stability, and behavioral context interact synergistically. This complexity poses inherent challenges for conventional randomized trial designs, but also constitutes a defining characteristic of the intervention.

Across the nine included trials, consistent improvements were observed in the principal outcomes assessed, particularly pain, mobility, fatigue, and quality of life, especially among individuals with fibromyalgia, osteoarthritis, and rheumatoid arthritis. These findings are consistent with previous systematic reviews on spa- and climate-based therapies (Bender et al. 2014; Forestier et al. 2017; Protano et al. 2023; Amatya et al. 2023) and are further supported by Antonelli and Donelli (2025), who also reported benefits in dermatological conditions.

From a biometeorological perspective, the observed clinical benefits of climatotherapy are plausibly mediated by combined atmospheric and environmental factors, including increased barometric pressure, altered solar radiation spectra, marine aerosol exposure, and thermohygrometric stability. These factors interact with physiological processes related to inflammation, circadian regulation, vitamin D metabolism, and autonomic balance, positioning climatotherapy as a complex biometeorological intervention rather than a purely rehabilitative modality.

The statistically inconclusive pooled effect for pain should therefore be interpreted as a methodological signal of imprecision rather than as evidence against climatotherapy, reinforcing the relevance of condition-specific analyses and narrative synthesis.

Despite substantial clinical heterogeneity, we were able to conduct a limited quantitative synthesis restricted to controlled trials. Single-study estimates indicated moderate short-term benefits for morning stiffness and pain, aligning with the direction of effects observed across trials. Outside the scope of this review, a recent meta-analysis on balneotherapy reported reductions in pain and improvements in quality of life, but with very low certainty of evidence due to risk of bias and suspected publication bias (Aribi et al. 2025). This contrast underscores that, even for therapies with apparent benefits, rigorous methodological standards are required. For climatotherapy and its dimensions (e.g., thalassotherapy, marine mud) in marine environments or the Dead Sea, our findings highlight the need for well-designed, standardized, and adequately powered trials.

Fibromyalgia and ankylosing spondylitis were the most frequently studied conditions, with evidence of reduced pain and fatigue, improved sleep, and transient psychological gains. These findings support the hypothesis that mineral-rich marine environments, combined with the thermal and mechanical effects of immersion, may influence pain perception and inflammation (Fioravanti et al. 2011). Antonelli and Donelli (2025) also described multifactorial mechanisms, including anti-inflammatory effects of marine minerals, vitamin D synthesis through sun exposure, and marine aerosols with potential respiratory benefits. Patients with osteoarthritis and rheumatoid arthritis also demonstrated gains in joint function and mobility, reinforcing the therapeutic role of climatotherapy as an adjunct to conventional care.

The interventions reviewed consistently demonstrated clinical benefits, but safety remains poorly documented. The lack of systematic reporting of adverse events across most trials is a recurrent limitation in climatotherapy and balneology research (Szendi et al. 2025). The apparent absence of serious complications may reflect a favorable safety profile, but it may also be due to underreporting or insufficient monitoring. This contrasts with broader literature on balneotherapy, in which adverse effects are usually mild and transient, such as skin irritation or fatigue (Fioravanti et al. 2011). Future trials should therefore include explicit safety endpoints, standardized reporting of adverse events, and long-term follow-up to establish a reliable safety profile.

Most interventions were multimodal, combining climatotherapy with exercise, relaxation, or education. This mirrors real-world rehabilitation practice but complicates the isolation of specific effects. Future research should consider arms focusing exclusively on climatotherapy or thalassotherapy to clarify their independent contribution.

Evidence from related aquatic interventions further supports these trends. In fibromyalgia, meta-analyses show that balneotherapy reduces pain and fatigue and improves sleep and well-being (Cao et al. 2021; García-López et al. 2024). Osteoarthritis trials also demonstrated functional improvements (Protano et al. 2023). Spa therapy combined with outdoor walking improved low back pain and mood (Huber et al. 2019). While not restricted to marine contexts, their mechanisms and outcomes are relevant to climatotherapy.

Structural barriers also hinder progress. Limited funding and absence of multicenter collaborations restrict protocol standardization and large-scale evidence generation (Szendi et al. 2025). As Lundh et al. (2017) warned, insufficient methodological rigor and reliance on non-independent funding risk undermining credibility in health policy contexts. Still, the persistence of research efforts despite scarce resources demonstrates resilience and creativity in the field.

Encouragingly, positive outcomes were observed across heterogeneous protocols, suggesting consistency of therapeutic effects. With methodological refinement, international collaboration, and geographic diversity, climatotherapy could consolidate as an accessible, evidence-based intervention of global relevance. Recent studies confirm its potential: García-López et al. (2024) found fibromyalgia benefits lasting up to six months, Protano et al. (2023) confirmed improvements in osteoarthritis, and Ferrara et al. (2025) reported anti-inflammatory and stress-reducing effects in post-COVID populations. Together, these findings reinforce climatotherapy’s integrative and multimodal potential.

Taken together, climatotherapy offers immediate symptom relief and multidimensional benefits, particularly for chronic pain patients insufficiently responsive to pharmacological therapy. Its integration into rehabilitation programs may reduce drug reliance and enhance psychosocial well-being. Beyond therapeutic promise, climatotherapy aligns with sustainable and patient-centered care models, underscoring its relevance in contemporary health agendas.

### Limitations of the meta-analytical approach

Only two studies met the eligibility criteria for quantitative pooling in the meta-analysis of pain outcomes. Although a random-effects model with Hartung-Knapp adjustment (Hartung and Knapp 2001) was applied, the resulting pooled estimate illustrates a very wide confidence interval (SMD = -0.47; 95% CI: -5.47 to 4.53), indicating substantial statistical imprecision.

This finding does not reflect a lack of effectiveness of climatotherapy as a therapeutic intervention, but rather the inherent methodological fragility of conducting meta-analyses with a very small number of studies (k = 2), particularly in the presence of clinical and methodological heterogeneity. In this review, the pooled studies differed markedly in clinical condition (osteoarthritis versus fibromyalgia), intervention context, and outcome variability, which limits the interpretability of a single global estimate.

In addition, publication bias and small-study effects could not be formally assessed due to the very limited number of included studies (k < 10), in accordance with Cochrane Handbook recommendations.

Importantly, the application of the Hartung-Knapp adjustment (Hartung and Knapp 2001), recommended for meta-analyses with few studies, further contributed to the widening of the confidence interval by appropriately accounting for between-study uncertainty. While this approach is more conservative, it avoids spuriously precise estimates that may arise from conventional methods in underpowered syntheses.

Additionally, the use of standardized mean differences for pain outcomes may amplify uncertainty when sample sizes are small and baseline variability differs substantially across studies. Under these conditions, the pooled estimate should be interpreted as statistically inconclusive rather than clinically contradictory.

The meta-analysis was included primarily to illustrate the methodological challenges inherent in climatotherapy research. Therefore, its pooled estimate should be interpreted as exploratory and hypothesis-generating rather than confirmatory and should be considered alongside the predefined subgroup analysis and the structured narrative synthesis.

### Methodological limitations of the included studies

The interpretation of the findings of this review must be considered in light of several methodological limitations inherent to the included studies.

Methodological limitations were recurrent. Poor reporting of randomization and allocation concealment increased the risk of selection bias (Sukenik et al. 1995; Elkayam et al. 2000; Andrade et al. 2008), a weakness also noted more broadly in balneology research. The lack of blinding of participants and therapists is particularly concerning for subjective outcomes such as pain and quality of life, as lack of blinding tends to inflate effects (Hróbjartsson et al. 2014). A recent scoping review confirmed this fragility: 74% of balneology trials did not report blinding, only 11% attempted double-blinding, and just one validated a placebo (Szendi et al. 2025). In addition, intention-to-treat analyses and systematic reporting of dropouts were often absent, raising risks of attrition bias (Higgins et al. 2022). Selective outcome reporting was also present, consistent with broader clinical research patterns (Chan et al. 2004).

Most studies scored intermediate quality on the PEDro Scale (range 2–8/10, typically 6/10), largely due to consistent failures in allocation concealment, intention-to-treat analysis, and blinding. This pattern reflects not only individual study weaknesses but also the methodological culture of the 1990–2000 s, when reporting standards such as CONSORT were not widely enforced. Two trials published as Brief Reports (Sukenik et al. 1999; Elkayam et al. 2000) further limited methodological detail. The complementary analysis (Table 5) highlighted that, while interventions were generally well described and multimodal elements reported, adverse events, therapist expertise, and adherence were rarely addressed. 

**Table 5:** Complementary indicators of methodological rigor and reporting quality of the included studies

Outcome	Condition	K	SMD (95% CI)	*p*-value	I²
Pain (Overall)	Mixed	2	-0.47 [-5.47; 4.53]	0.4438	59.9%
Pain (Subgroup)	Osteoarthritis	1	-1.01 [-1.98; -0.04]	< 0.05	N/A
Pain (Subgroup)	Fibromyalgia	1	-0.18 [-0.53; 0.16]	0.158	N/A
Depression (BDI)	Fibromyalgia	1	-0.09 [-0.43; 0.25]	0.5968	N/A
Tender Points	Fibromyalgia	1	-0.50 [-0.85; -0.15]	0.0047	N/A
SF-36 PCS (Physical)	Fibromyalgia	1	0.36 [0.02; 0.71]	0.0391	N/A
SF-36 MCS (Mental)	Fibromyalgia	1	-0.04 [-0.38; 0.30]	0.8206	N/A
FIQ Total Score	Fibromyalgia	1	-0.33 [-0.67; 0.02]	0.0612	N/A

### Methodological roadmap for future climatotherapy trials

Future clinical trials in climatotherapy should move beyond descriptive reporting and adopt standardized methodological strategies to improve comparability and cumulative evidence generation. First, to mitigate performance bias inherent to non-blinded interventions, future studies should prioritize credible active control conditions, such as land-based rehabilitation programs matched for duration and intensity, or saline water with minimal mineral concentration.

Second, the development of a core outcome set for climatotherapy trials is strongly recommended. At a minimum, standardized assessment of pain (e.g., VAS), physical function, and health-related quality of life (e.g., SF-36), measured at baseline, post-intervention, and at a minimum six-month follow-up, would substantially enhance cross-study comparability and enable robust meta-analyses.

Finally, systematic reporting of adverse events, including mild and transient effects, should become standard practice to establish a reliable safety profile and support clinical and policy decision-making. The apparent absence of serious adverse events should be interpreted with caution, as it likely reflects insufficient and inconsistent reporting rather than confirmed safety. Future trials should therefore treat safety assessment as a core outcome, rather than a secondary or implicit assumption.

## Conclusion

This review shows that climatotherapy, across its various modalities, provides consistent short-term benefits for chronic musculoskeletal and rheumatologic conditions (reducing pain and improving mobility, physical function, and quality of life), even in trials conducted with limited resources. Where controlled comparisons were available, condition-specific estimates indicated a clinically meaningful effect on pain, most clearly in osteoarthritis, whereas fibromyalgia showed smaller and less certain effects. No serious adverse events were reported, but safety was inconsistently assessed, so tolerability cannot yet be firmly established.

These findings support the careful consideration of climatotherapy as a complementary option for selected patients, particularly those with osteoarthritis or chronic musculoskeletal pain refractory to conventional care, within shared decision-making and transparent communication about the transient nature of the benefits. Consolidating its role will require multicenter randomized trials with standardized protocols, core outcome sets, and systematic safety reporting. From a health-policy perspective, the reproducibility of short-term benefits across diverse settings justifies piloting climatotherapy programs within public health systems, coupled with rigorous evaluation of cost-effectiveness and sustainability.

## Data Availability

All data extracted from the studies included in this review, as well as the data extraction forms and the completed PRISMA checklist, are available from the corresponding author upon reasonable request. No new datasets were generated. A narrative synthesis was conducted for all trials, and a limited quantitative synthesis was performed for outcomes with comparable controlled data. The dataset extracted from included studies, the meta-analysis outputs (tables/figures), and the PRISMA 2020 checklist are available from the corresponding author upon reasonable request. Analysis scripts and R session information can also be provided.

## Appendix 1

Ovid MEDLINE(R) ALL < 1946 to Dec 26, 2023>.

1 exp musculoskeletal diseases/ or rheumatology/ or exp orthopedics/ or orthopedic procedures/ 1,274,821.

2 (musculoskeletal or arthriti$ or orthop? edic? or osteo$ or polymyalg$ or periarthrit$).ti. 430,443.

3 (arthritis or back pain or chondrocalcinosis or dermatomyositis or dupuytren? contracture or fibromyal$ or Fibrositis or Fibrositides or gout or hyperostos$ or low$ back or lupus or osteitis or osteoarthrit$ or osteoarthrop$ or osteochondr$ or Osteonecros$ or osteoporos$ or periarthriti$ or polymyalgia? or raynaud disease? or rheumatism or rheumatic disease? or sciatica or scleroderma$ or Spondylarthrit$).ti, ab. 613,727.

4 (((cartiledge or connective tissue? or joint? or ligament? or muscula$ or myofascial or neck or soft tissue? or spine or spinal) adj2 (damage? or disease? or disorder? or injury or injuries or pain? or strain? )) and (care or treatment)).ti. 5310.

5 (athletic? adj2 (strain? or injury or injuries)).ti. 528.

6 Dermatomyositis/ or Dupuytren’s Contracture/ or Lupus Erythematosus, Cutaneous/ or Lupus Erythematosus, Systemic/ or exp back pain/ or neck pain/ or sciatica/ or exp Raynaud Disease/ or exp Scleroderma, Systemic/ or exp arm injuries/ or athletic injuries/ or exp back injuries/ or exp dislocations/ or exp fractures bone/ or fractures cartilage/ or exp hand injuries/ or exp hip injuries/ or exp leg injuries/ or multiple trauma/ or exp neck injuries/ or soft tissue injuries/ or exp spinal cord injuries/ or exp spinal injuries/ or exp “sprains and strains”/ or exp tendon injuries/ or exp musculoskeletal system/ 1,991,467.

7 or/1–6 2,967,024 [--------------MED].

8 Climatotherapy/ or Thalassotherapy/ or (Thalassotherap* or “climate therapy” or Climatotherap* or “Dead Sea” or “thermal water”).ti, ab. 1771 [-------Climatotherapy].

9 7 and 8 142.

Cochrane.

[mh Climatotherapy] or [mh Thalassotherapy] or (Thalassotherap* or “climate therapy” or “Dead Sea” or “thermal water” or Climatotherap*):ti, ab.

= 134.

Pedro.

thalassotherap* OR “climate therapy” OR climatotherap* OR “Dead Sea” OR “thermal water”.

subdiscpline: Musculoskeletal.

= 19.

## Appendix 2

**Table:**

Author/Year	Study	Reason for Exclusion
Tsvetanov et al. 1962	Changes in non-specific immuno-biological reactivity in patients with osteoarticular tuberculosis under the influence of thalasso therapy	Not a clinical trial (randomized or non-randomized)
Sukenik et al. 1992	Mud pack therapy in rheumatoid arthritis	Not using climatotherapy/thalassotherapy in marine/Dead Sea
Sukenik et al. 2001	Balneotherapy at the Dead Sea area for patients with psoriatic arthritis and concomitant fibromyalgia	Only sulfurous water was used
Machtey 2009	Dead Sea and Dead Sea salt balneotherapy for arthritis	Not a clinical trial (randomized or non-randomized)
Abu-Shakra et al.,2014	Dead Sea mud packs for chronic low back pain	Not using climatotherapy/thalassotherapy in marine/Dead Sea
2023 (Trial ID: KCT0008081)	The Effects of a Marine Healing Program on Chronic Pain and Stress	Not a clinical trial (randomized or non-randomized)

## Appendix 3

### Detailed Methods

This appendix presents the detailed methodological descriptions condensed in the main text (Methods), relocated here at the reviewers’ suggestion to improve readability while preserving full transparency.

### Eligibility criteria (detailed)

The inclusion criteria were as follows: (1) Clinical trials and randomized controlled trials; (2) Studies addressing musculoskeletal disorders; (3) Studies utilizing climatotherapy or any of its dimensions (thalassotherapy, marine mud, etc.) in a marine environment or the Dead Sea; (4) Studies with outcomes including pain reduction, improvement in quality of life, or other clinically relevant parameters such as biomarkers, morning stiffness, functional limitations, and reduction in medication use.

The exclusion criteria were as follows: (1) literature reviews or studies based on secondary data were excluded, as well as any other studies that were not clinical trials (randomized or non-randomized); (2) studies that did not employ climatotherapy or any of its dimensions (e.g., thalassotherapy, marine mud) in a marine environment or the Dead Sea; (3) studies in which all intervention and control groups used sulfurous water; (4) articles for which full-text access was unavailable, even after attempts to contact the authors by email.

### Study selection (detailed)

All identified records were imported into Rayyan, where the primary researcher removed duplicates manually. Study selection was then performed independently and blinded by two reviewers.

Titles and abstracts were screened against the predefined inclusion and exclusion criteria, and records meeting them advanced to full-text review.

When full texts were unavailable, authors were contacted by email (two messages two weeks apart, with a further two-week wait before exclusion). Disagreements at any stage were resolved by consensus or, when necessary, by a third reviewer.

### Data extraction (detailed)

Outcomes of interest included both primary and secondary endpoints, such as pain, fatigue, mobility, quality of life, and other clinically relevant measures. Whenever available, we recorded the instruments used (e.g., VAS, SF-36, FIQ), numerical results, follow-up time points, statistical significance, and authors’ conclusions. Complementary information was also extracted to support interpretation, including multimodal elements (e.g., exercise, relaxation, education), adherence, therapist expertise, and adverse events. These indicators were qualitatively integrated into the overall synthesis, complementing the quantitative results by contextualizing the interventions and their practical feasibility, rather than being treated as separate statistical outcomes.

All extracted data were organized into a structured summary table (Table 1). The assessment of methodological quality was performed independently using the PEDro Scale, as detailed in the “Risk of bias” section. In studies with multiple intervention arms, when some used sulfurous water and others did not, only the eligible arms were considered according to the predefined criteria. Thus, data from non-sulfurous interventions were included, while those involving sulfurous water were disregarded.

Missing or unclear data were reported narratively based on information available in the original publications. No data imputation, estimation, or conversion procedures were applied.

### Risk of bias assessment (detailed)

In addition to the PEDro assessment, complementary indicators of methodological rigor and reporting quality – such as clarity of intervention description, therapist expertise, participant adherence, and inclusion of multimodal components (e.g., exercise, relaxation, education) – were qualitatively examined. These indicators are summarized in Table 5 and were considered in the overall appraisal of trial quality, as discussed in the Results and Discussion sections.

This integrated approach allowed a contextualized evaluation of methodological strengths and limitations specific to climatotherapy research. All assessments were performed by the lead researcher and independently verified by a second reviewer (FH).

Although the PEDro scale was originally developed for randomized controlled trials, it was applied to all included clinical trials to allow a standardized appraisal of key methodological domains. Scores from the non-randomized study were interpreted descriptively and not directly compared with those of randomized trials.

### Data synthesis (detailed)

A structured narrative synthesis was conducted for all eligible studies, and quantitative synthesis was restricted to randomized or non-randomized controlled trials with between-group comparisons. Continuous outcomes were summarized as standardized mean differences (Hedges’ g) with 95% confidence intervals. Random-effects models using restricted maximum likelihood (REML) were applied when at least two studies (k ≥ 2) contributed data; when only one study was available (k = 1), effect sizes were reported descriptively without pooling. For outcomes where lower scores indicate improvement (e.g., pain VAS, morning stiffness), negative SMD values favored the intervention.

Statistical heterogeneity (τ² and I²) was calculated and reported when quantitative synthesis was feasible (k ≥ 2). Given the limited number of trials, quantitative analyses were included to illustrate the magnitude and uncertainty of effects under real-world constraints rather than to provide definitive pooled estimates.

Clinical and methodological heterogeneity, including variation in intervention duration, frequency, multimodal components, outcome measures, and sample size) was examined qualitatively across studies. No sensitivity analyses or meta-regressions were conducted due to the small number of available trials. A predefined subgroup analysis by clinical condition was performed only when sufficient controlled data were available (k ≥ 2) to aid interpretation of heterogeneity.

The strength of evidence was judged qualitatively based on consistency in the direction of effects and methodological quality, with findings interpreted as stronger when consistent across higher-quality studies and weaker when inconsistent or derived from single-study estimates. Publication bias and small-study effects were not formally assessed because fewer than ten studies contributed to the quantitative synthesis, in accordance with Cochrane Handbook recommendations (Higgins et al. 2022).

## Declarations

### Standards of Reporting

This review followed the PRISMA 2020 guidelines.

### Use of Artificial Intelligence

During the preparation of this manuscript, the authors used ChatGPT (OpenAI) and Claude (Anthropic) for language editing and translation. All content was reviewed and edited by the authors, who take full responsibility for the final version of the manuscript.
